# Effects of nilotinib on regulatory T cells: the dose matters

**DOI:** 10.1186/1476-4598-9-22

**Published:** 2010-01-29

**Authors:** Fei Fei, Yingzhe Yu, Anita Schmitt, Markus T Rojewski, Baoan Chen, Jochen Greiner, Marlies Götz, Donald Bunjes, Michael Schmitt

**Affiliations:** 1 Department of Internal Medicine III, University of Ulm, 89081 Ulm, Germany; 2 Institute for Transfusion Medicine, University of Ulm, and Institute for Clinical Transfusion Medicine and Immunogenetics Ulm gemeinnuetzige GmbH, 89081 Ulm, Germany; 3 Department of Hematology and Oncology, ZhongDa Hospital, Southeast University, Nanjing 210009, PR China; 4 Current address: Center for Internal Medicine, Medical Clinic III - Department of Hematology, Oncology, Palliative Medicine, University of Rostock, Ernst-Heydemann-Str 6, 18057 Rostock, Germany

## Abstract

**Background:**

Nilotinib is a tyrosine kinase inhibitor with high target specificity. Here, we characterized the effects of nilotinib for the first time on CD4^+^CD25^+ ^regulatory T cells (Tregs) which regulate anti-tumor/leukemia immune responses.

**Design and Methods:**

Carboxyfluorescein diacetate succinimidyl ester (CFSE) and 5-bromo-2-deoxy -uridine (BrdU) were used to assess the proliferation and cell cycle distribution of Tregs. The expression of the transcription factor forkhead box P3 (FoxP3) and the glucocorticoid-induced tumor necrosis factor receptor (GITR) were measured by flow cytometry. Western blotting analysis was used to detect the effects of nilotinib on the signal transduction cascade of T-cell receptor (TCR) in Tregs.

**Results:**

Nilotinib inhibited the proliferation and suppressive capacity of Tregs in a dose-dependent manner. However, the production of cytokines secreted by Tregs and CD4^+^CD25^- ^T cells was only inhibited at high concentrations of nilotinib exceeding the mean therapeutic serum concentrations of the drug in patients. Only high doses of nilotinib arrested both Tregs and CD4^+^CD25^- ^T cells in the G_0_/G_1 _phase and down-regulated the expression of FoxP3 and GITR. In western blotting analysis, nilotinib did not show significant inhibitory effects on TCR signaling events in Tregs and CD4^+^CD25^- ^T cells.

**Conclusions:**

These findings indicate that nilotinib does not hamper the function of Tregs at clinical relevant doses, while long-term administration of nilotinib still needs to be investigated.

## Introduction

Nilotinib (AMN107, Tasigna; Novartis Pharma, Basel, Switzerland) is a new, orally active, selective inhibitor of the ABL/BCR-ABL, CSF-1R, DDR, KIT, and PDGFR tyrosine kinases, that is more potent against chronic myeloid leukemia (CML) cells *in vitro *than is imatinib. Like imatinib, nilotinib acts through competitive inhibition at the ATP-binding site of BCR-ABL, leading to the inhibition of tyrosine phosphorylation of proteins that are involved in the intracellular signal transduction mediated BCR-ABL. Nilotinib has a higher binding affinity and selectivity for the ABL kinase than does imatinib, which translates into 20- to 50-fold greater inhibitory activity than imatinib in imatinib-sensitive CML cells and 3- to 7-times the activity in imatinib-resistant cell lines with mutant ABL kinases [1-5]. Results from a phase I dose escalation study performed in patients with imatinib-resistant CML and Philadelphia (Ph) chromosome-positive acute lymphoblastic leukemia (ALL) indicated that nilotinib produced significant hematologic and cytogenetic responses in all phases of CML [2]. Furthermore, 400 mg nilotinib was administered orally twice daily, proved to be very active and safe in phase II study of patients with chronic phase CML and accelerated-phase CML post-imatinib resistance and intolerance [6,7]. Now, clinical trials with nilotinib are ongoing in patients with imatinib-resistant or imatinib-intolerant accelerated-phase CML and Ph-positive ALL [7,8].

Naturally arising CD4^+^CD25^+ ^regulatory T cells (Tregs) have the potential to suppress aberrant immune responses and to regulate peripheral T-cell homeostasis [9]. Tregs play a crucial role in both the induction and maintenance of tolerance. This active immune regulation may contribute not only to the control of immune responses to self-antigens, thereby preventing autoimmune diseases, but also the control of responses to non-self molecules in adaptive immunity. Numerous experimental and clinical studies indicate that manipulating the balance between regulatory and effector T cells is an effective strategy to control immune responsiveness after transplantation. Therefore a better understanding of regulatory T cells biology is essential for exploiting this strategy to clinical therapy [10].

There is evidence that imatinib and dasatinib have inhibitory effects on immune reconstitution and T cell proliferation and function [11-15]. Furthermore, nilotinib was shown to have an inhibitory effect on CD8^+ ^T cells *in vitro *[16], however little is known about its effects on Tregs [17]. Therefore, we wondered to what extent and by which mechanisms nilotinib affects the immune system, particularly for Tregs. In this study, we examined the effects of nilotinib on both Tregs and CD4^+^CD25^- ^T cells. We indicate that nilotinib similarly inhibits proliferation and function of Tregs as well as CD4^+^CD25^- ^T cells only at high concentrations greater than 10 μM nilotinib which exceeds the therapeutic range achieved with current standard dosing schedules.

## Design and Methods

### Nilotinib, imatinib and dasatinib

Nilotinib and imatinib were provided by Novartis Pharmaceuticals, Basel, Switzerland. Dasatinib was purchased from Bristol-Myers Squibb, New York, NY, USA and stored in aliquots at -20°C as 10 mM stock solution in DMSO.

### Cell isolation and culture

Peripheral blood mononuclear cells (PBMCs) were isolated by Ficoll-Biocoll Separation Solution (Biochrom, Berlin, Germany) as described previously [18]. CD4^+^CD25^+ ^T cells and CD4^+^CD25^- ^T cells were selected from the total PBMCs using CD4^+^CD25^+ ^regulatory T cell isolation kit (Miltenyi Biotec, Bergisch Gladbach, Germany), according to the manufacturer's instruction. This procedure led to the complete positive selection of CD4^+^CD25^+ ^T cells (purity ≥ 90%), and negative depletion of CD4^+^CD25^- ^T cells, as measured by flow cytometry (FACSan, Becton Dickinson, Franklin Lake, NJ, USA). Cells were cultured in RPMI 1640 (Biochrom AG, Berlin, Germany) supplemented with 10% human AB serum (Germany Red Cross Blood Center, Ulm, Germany), 2 mM L-glutamine and 100 units/ml penicillin-streptomycin (Invitrogen Gibco, Grand Island, USA).

### CFSE-based cell proliferation

Isolated CD4^+^CD25^+ ^T cells (1 × 10^6^/ml) and CD4^+^CD25^- ^T cells (1 × 10^6^/ml) were labeled with 0.5 μM vital dye carboxyfluorescein diacetate succinimidyl ester (CFSE, Invitrogen Gibco, Grand Island, USA) just before stimulation. Labeled CD4^+^CD25^+ ^T cells (1 × 10^5^/well) or CD4^+^CD25^- ^T cells (1 × 10^5^/well) were stimulated with anti-CD3 (OKT3; eBioscience, San Diego, CA, USA) and 2 μg/ml soluble anti-CD28 (CD28.2, BD Pharmingen™, Heidelberg, Germany). 300 units/ml IL-2 was used to expand CD4^+^CD25^+ ^T cells. After 4 days of stimulation, cell division was monitored by levels of CFSE dilution. Unstimulated T cells served as negative control in all experiments.

### Suppression assay

CD4+CD25^+ ^T cells were incubated for 4 days with CFSE^+^-labeled CD4+CD25^- ^T cells, with each population 5 × 10^4 ^cells in the presence of anti-CD3 and anti-CD28. In some experiments, CD4^+^CD25^+ ^T cells were first incubated with nilotinib overnight, then the cells were washed for three times and co-cultured with CFSE^+^-labeled CD4^+^CD25^- ^T cells as a ratio of 1:1 as mentioned above.

### Apoptosis assay

CD4^+^CD25^+ ^T cells and CD4^+^CD25^- ^T cells were treated with nilotinib for 48 h. Cells were harvested and stained with Annexin V* fluorescein isothiocyanate (FITC) and propidium iodide (PI) (Annexin V*FITC apoptosis detection kit I; BD Pharmingen™, Heidelberg, Germany). Apoptotic cells were defined by flow cytometry as Annexin V positive and PI-negative cells.

### Cell cycle analysis

An indirect 5-bromo-2-deoxyuridine (BrdU)-FITC flow kit (BD Pharmingen™, Heidelberg, Germany) was used to determine the cycle kinetics of CD4^+^CD25^+ ^T cells and CD4^+^CD25^- ^T cells, and to measure the incorporation of BrdU into the DNA of proliferating cells. CD4^+^CD25^+ ^T cells or CD4^+^CD25^- ^T cells were treated with different concentrations of nilotinib as indicated for 4 days. Cells were harvested and measured according to the manufacturer's instruction.

### Cytokine analysis

CD4^+^CD25^+ ^T cells and CD4^+^CD25^- ^T cells were stimulated with anti-CD3, anti-CD28 and IL-2 in the presence or absence of 25 μM nilotinib. After 4 days incubation, supernatants were collected and analyzed for cytokines according to the instruction of Proteome Profiler Array (R&D Systems, Minneapolis, MN, USA).

### Flow cytometry

Cells were phenotyped by 4- or 5- color Abs and measured by flow cytometry as described previously [18]. The following conjugated Abs were used: CD4* fluorescein isothiocyanate (FITC), CD4*phycoerythrin-Cyanine 7 (PE-Cy7), CD25* phycoerythrin-Cyanine 5 (PE-Cy5), CD25* Allophycocyanin (APC) (BD Pharmingen™, Heidelberg, Germany), transcription factor forkhead box P3 (FoxP3)*phycoerythrin (PE) (PCH 101; eBioscience, San Diego, CA), and glucocorticoid-induced tumor necrosis factor receptor (GITR)*PE (R&D Systems, Minneapolis, MN, USA),

### Western blotting

CD4^+^CD25^+ ^T cells, CD4^+^CD25^- ^T cells or Jurkat T cells (1 × 10^6 ^cells/well) were treated with different concentrations of imatinib, nilotinib or dasatinib for 1 hour and stimulated with anti-CD3/CD28 for 15 minutes. Western blotting analysis was performed as previously described [18] by using the following antibodies: phospho-Lck, Lck, phospho-ZAP-70, ZAP-70, phospho-p44/42 MAP kinase, p44/42 MAP kinase, phospho-Akt, Akt, src, phospho-src family (Tyr416), non-phospho-src (Tyr416), phospho-src (Tyr527), non-phospho-src (Tyr527), phospho-NF-κB p65 (Ser536)(93H1) and NF-κB p65 from Cell Signaling Technology, USA and anti-Actin C-11 (Santa Cruz Biotechnology, Heidelberg, Germany) to confirm equal protein loading.

### Statistical analysis

Statistical analysis was performed with SPSS software. Data were presented as mean ± standard deviation (SD). Statistical significance of differences between groups was evaluated using one-way-ANOVA (post hoc Scheffe test). The value of *P *< .05 was considered to be statistically significant.

## Results

In phase I clinical studies, at the steady-state, mean serum levels of nilotinib were: 1.0 μM at 400 mg once daily, 1.7 μM at 400 mg twice daily, and 2.3 μM at 600 mg twice daily; at 400 mg twice daily, the dose selected for phase II studies, steady state mean serum peak levels of drug were 3.6 μM [2]. Based upon this, to include the clinically relevant dose range of nilotinib, all assays described in this manuscript were performed at concentrations between 1 and 25 μM.

### Nilotinib inhibits the proliferation of CD4^+^CD25^+ ^T cells and CD4^+^CD25^- ^T cells in a dose-dependent manner

**Figure 1:**
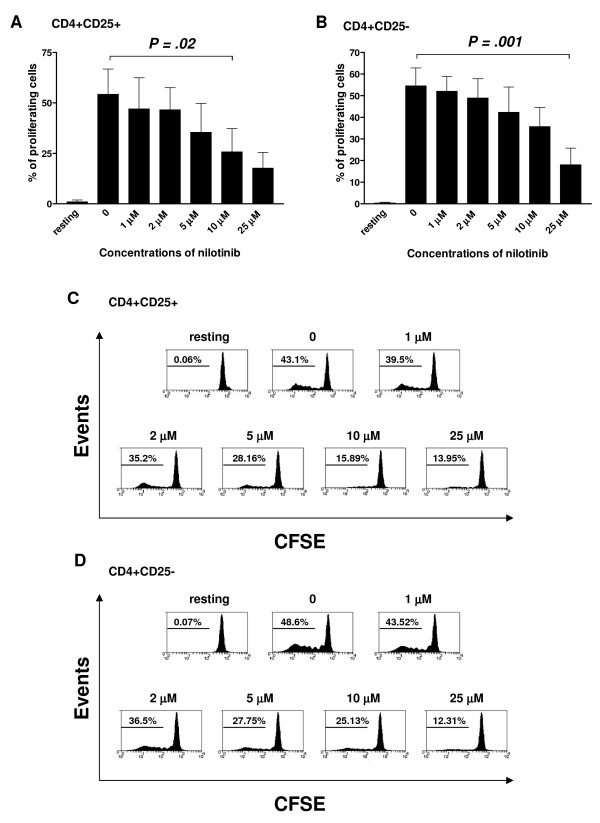
**Nilotinib inhibits the proliferation of CD4^+^CD25^+ ^T cells and CD4^+^CD25^- ^T cells in a dose-dependent manner**. CD4^+^CD25^+ ^T cells or CD4+CD25^- ^T cells were labeled with CFSE and cultured with different concentrations of nilotinib as indicated. After 4 days incubation, cells were harvested and stained with CD25 and FoxP3. Cell division was assessed by the dilution of CFSE. Panels A and B show the combined results from four independent experiments with similar results. Displayed are mean values ± SD. Panels C and D display the results of a representative experiment.

Human CD4^+^CD25^+ ^T cells could be expanded by anti-CD3, anti-CD28 and high doses of IL-2 (Figure 1A and 1C), in accordance with previous findings [19,20]. Addition of nilotinib inhibited the proliferation of both CD4^+^CD25^+ ^T cells (Figure 1A and 1C) and CD4^+^CD25^-^T cells (Figure 1B and 1D) in a dose-dependent manner as shown in Figure 1. The effect was significant at concentrations of nilotinib greater than 10 μM and 25 μM for CD4^+^CD25^+ ^T cells and CD4^+^CD25^- ^T cells respectively. However the concentrations exceeded the mean serum levels achieved in patients to whom nilotinib was administered.

### Nilotinib has no inhibitory effect on the suppressive capacity of CD4^+^CD25^+ ^T cells

**Figure 2:**
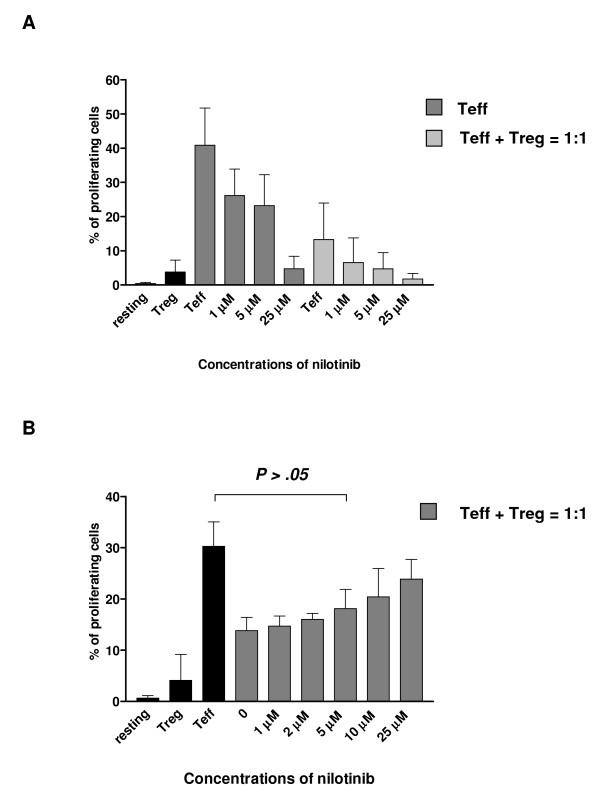
**Nilotinib does not hamper immunosuppressive capacity of CD4^+^CD25^+ ^T cells at clinical relevant doses**. Panel A: Purified CD4^+^CD25^+ ^T cells or CD4^+^CD25^- ^T cells (Teff) were labeled with CFSE. CFSE^+^-labeled CD4^+^CD25^- ^T cells were stimulated with anti-CD3 and anti-CD28 in the presence or absence of nilotinib. The proliferation of CD4^+^CD25^- ^T cells was measured by the division of CFSE. Combined results from three independent experiments were shown. Panel B: CD4^+^CD25^+ ^T cells were first incubated with different concentrations of nilotinib overnight in the presence of IL-2. After washing cells for three times, CD4^+^CD25^+ ^T cells were co-cultured with CFSE^+^-labeled CD4^+^CD25^- ^T cells as a ratio of 1:1. Cell division was assessed by levels of CFSE dilution.

Our dose-dependent proliferation assays indicated that nilotinib at a concentration between 1-25 μM is suboptimal for the inhibition of the CD4^+^CD25^+ ^T cells and CD4^+^CD25^- ^T cells. Therefore, the use of these concentrations of nilotinib would allow us to assess an additional inhibition of CD4^+^CD25^- ^T cells by adding CD4^+^CD25^+ ^T cells. We observed that adding nilotinib to the co-cultures led to an additional inhibition of CD4^+^CD25^- ^T cells proliferation (Figure 2A) which might be the reason that nilotinib had a inhibitory effect on CD4^+^CD25^- ^T cells. Nilotinib at a concentration up to 25 μM did not hamper the suppressive capacity of CD4^+^CD25^+ ^T cell.

To further investigate the effect of nilotinib on the suppressive capacity of CD4^+^CD25^+ ^T cells, CD4^+^CD25^+ ^T cells were first incubated with or without different concentrations of nilotinib overnight in the presence of IL-2. Then, cells were washed three times to remove nilotinib and co-cultured together with CFSE^+^-labeled CD4^+^CD25^- ^T cells at a 1:1 ratio. After four days incubation, the proliferation of CD4^+^CD25^- ^T cells was measured by flow cytometry. To clarify whether CD4^+^CD25^+ ^T cells viability could be reduced by overnight incubation, we first measured the viability of CD4^+^CD25^+ ^T cells and CD4^+^CD25^- ^T cells after overnight incubation by staining cells with Annexin V*FITC and 7-AAD. Nilotinib did not reduce the viability of CD4^+^CD25^+ ^T cells and CD4^+^CD25^- ^T cells after pre-incubation overnight (data not shown). As shown in Figure 2B, nilotinib inhibited the suppressive capacity of CD4^+^CD25^+ ^T cells at concentrations higher than 5 μM.

### Nilotinib does not induce apoptosis of CD4^+^CD25^+ ^T cells or CD4^+^CD25^- ^T cells

**Figure 3:**
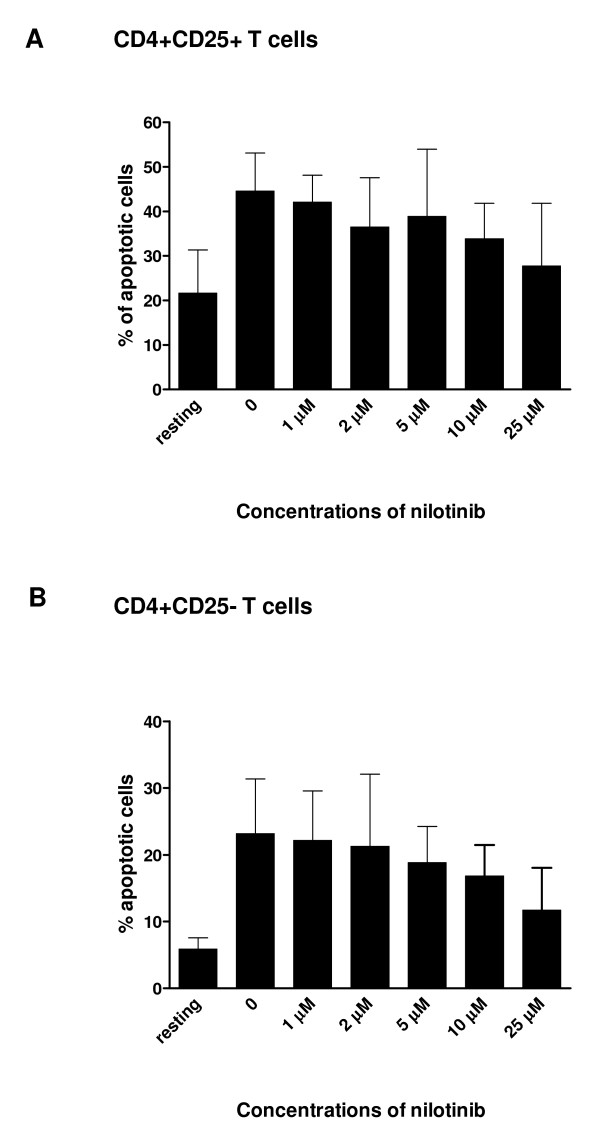
**Nilotinib does not induce apoptosis on CD4^+^CD25^+ ^T cells and CD4^+^CD25^-^T cells**. CD4^+^CD25^+ ^T cells (Panel A) and CD4^+^CD25^-^T cells (Panel B) were stimulated with anti-CD3, anti-CD28 and IL-2 with different concentrations of nilotinib as indicated for 48 h. Cells were then harvested and stained with Annexin V* FITC and PI. Apoptotic cells were defined by flow cytometry as Annexin V positive and PI negative cells.

To assess whether nilotinib might induce apoptosis or cell death in CD4^+^CD25^+ ^T cells and CD4^+^CD25^- ^T cells. We incubated CD4^+^CD25^+ ^T cells or CD4^+^CD25^- ^T cells with different concentrations of nilotinib as indicated for 4 days and measured apoptosis and cell death by Annexin V*FITC and PI staining. The percentage of Annexin V*FITC-positive and PI-negative events of CD4^+^CD25^+ ^T cells, which corresponds to apoptotic cells, remained between 27.75 ± 14.05% to 42.05 ± 6.09% compared with 44.53 ± 8.60% of untreated cells (*P *> .05) (Figure 3). The percentage of apoptotic CD4^+^CD25^- ^T cells remained between 16.80 ± 4.70% to 22.15 ± 7.41% compared with 23.18 ± 8.20% of untreated cells. According to apoptosis assay, CD4^+^CD25^+ ^T cells are much more sensitive to apoptosis than CD4^+^CD25^- ^T cells in agreement with the characteristics described previously for Tregs [21].

### Nilotinib arrests CD4^+^CD25^+ ^T cells and CD4^+^CD25^- ^T cells accumulating in the G_0_/G_1 _phase at high concentrations

Since many cytotoxic drugs are effective through inducing cell death, but also by causing an arrest in specific phases of cell cycle, we next examined whether nilotinib had an effect on cell cycle distribution. We stimulated CD4^+^CD25^+ ^T cells or CD4^+^CD25^- ^T cells with anti-CD3, anti-CD28 and IL-2 and measured cell cycle distribution by BrdU staining. Anti-CD3 and anti-CD28, in combination with IL-2, stimulated DNA synthesis in both CD4^+^CD25^+ ^T cells and CD4^+^CD25^- ^T cells and progressed cells into S phase, this effect was significantly inhibited by nilotinib at concentrations > 10 μM (data not shown), while nilotinib showed little significant inhibitory effect on cell cycle distribution at concentrations within the therapeutic dose range of the drug.

### Nilotinib shows inhibitory effects on cytokine secretion of CD4^+^CD25^+ ^T cells and CD4^+^CD25^- ^T cells

**Figure 4:**
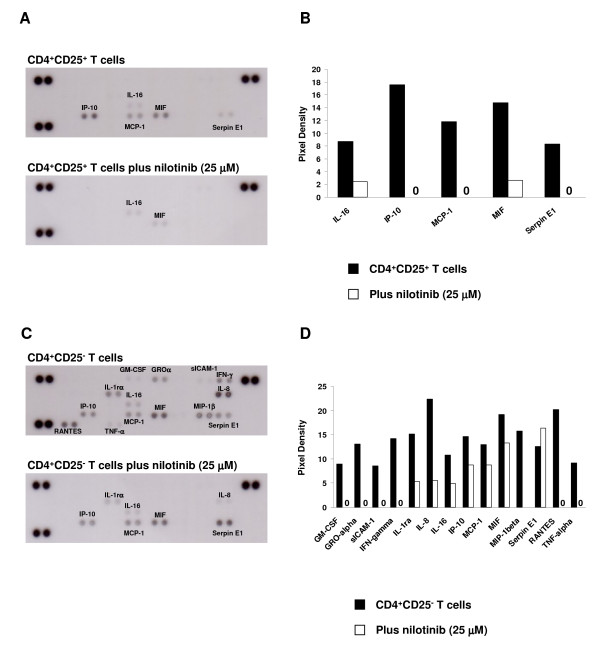
**Nilotinib inhibits the cytokine production by CD4^+^CD25^+ ^T cells and CD4^+^CD25^- ^T cells**. Panel A: CD4^+^CD25^+ ^T cells were stimulated with anti-CD3, anti-CD28 and IL-2 in the presence or absence of 25 μM nilotinib. After 4 days incubation, supernatants were collected for cytokine analysis. The high intensity spots in the three corners are positive controls, the lower right contains the negative controls. Panel B: Graphs of the relative density (subtracted the average background signals) are shown for selected cytokines. Array images are shown in panel A and profiles created by quantifying the background-subtracted mean spot pixel densities are identified as shown in Panel B by using image analysis software. Panel C + D: The cytokine production of CD4^+^CD25^- ^T cells treated with nilotinib was analyzed by above methods.

Cytokine production and release is a key process by which activated T cells participate in immune responses; and in situations of aberrant immune activity (autoimmunity, GVHD and transplant rejection) cytokines are involved in the pathophysiologic sequelae [22-24]. CD4^+^CD25^+ ^T cells and CD4^+^CD25^- ^T cells were stimulated and treated with or without 25 μM nilotinib. After 4 days of incubation, supernatants were collected for cytokine analysis (Figure 4). Nilotinib inhibited multiple cytokines production including pro-inflammatory cytokines (TNF-α, IFN-γ, and IL-1rα) and chemotactic factors (MIP-1β, MCP-1, RANTES, IP-10, etc) by CD4^+^CD25^- ^T cells at a high concentration of 25 μM. CD4^+^CD25^+ ^T cells only secreted few cytokines compared with CD4^+^CD25^- ^T cells (Figure 4C and 4D). T-cell receptor (TCR)-mediated activation Tregs were proved to have very low *in vitro *proliferative capacity and they do not produce cytokines compared with other T cells [25]. However, nilotinib inhibits the cytokines production by CD4^+^CD25^+ ^T cells significantly at a concentration of 25 μM.

### Nilotinib down-regulates the expression of CD4^+^CD25^+ ^T cell-specific molecules in a dose-dependent manner

**Figure 5:**
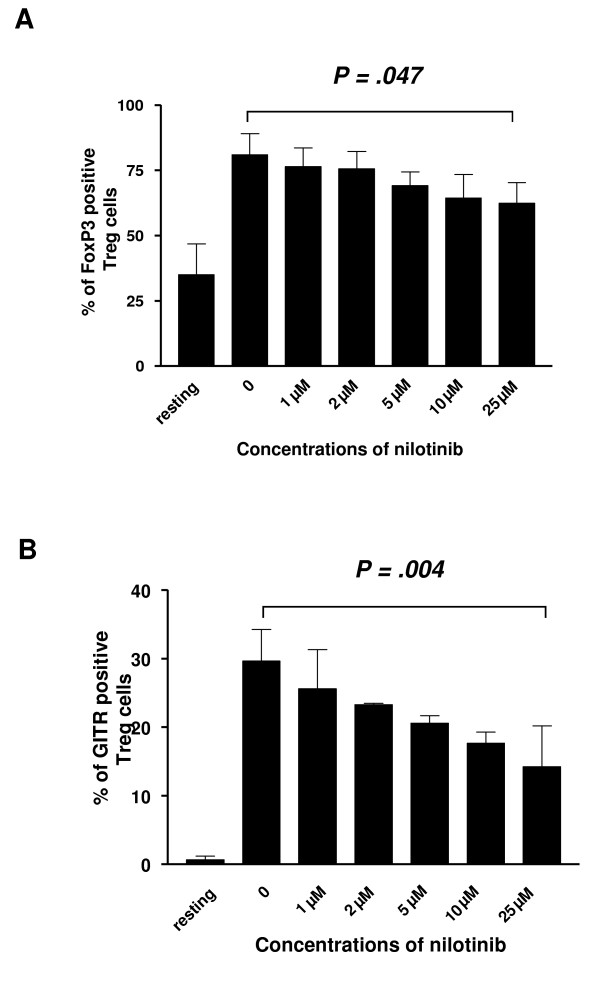
**High concentrations of nilotinib down-regulate the expression of FoxP3 and GITR by CD4^+^CD25^+ ^T cells**. Purified CD4^+^CD25^+ ^T cells were unstimulated or stimulated with anti-CD3, anti-CD28 and IL-2 in the presence or absence of nilotinib as indicated. Then cells were stained with FoxP3*PE or GITR*PE according to the manufacturer's instruction for fixation, permeabilization, and staining, after cells were stained for surface expression of CD4 and CD25. Nilotinib significantly decreased the intracellular expression of FoxP3 and GITR on CD4^+^CD25^+ ^T cells in a dose-dependent manner at concentrations of 25 μM. Data are representative of four independent experiments with similar results. Displayed are mean values ± SD.

To investigate the inhibitory effect of nilotinib on the phenotype of CD4^+^CD25^high ^T cells, CD4^+^CD25^+ ^T cells were stimulated with anti-CD3, anti-CD28 and IL-2 for 4 days. After stimulation, the expression of FoxP3 and GITR were up-regulated compared with unstimulated cells. However, nilotinib only at a concentration of 25 μM could down-regulate the expression of FoxP3 (P = .047) and GITR (P = .004) significantly (Figure 5) which was associated with the data in proliferation assay.

### Nilotinib does not show signaling events on TCR in CD4^+^CD25^+ ^T cells and CD4^+^CD25^- ^T cells

**Figure 6:**
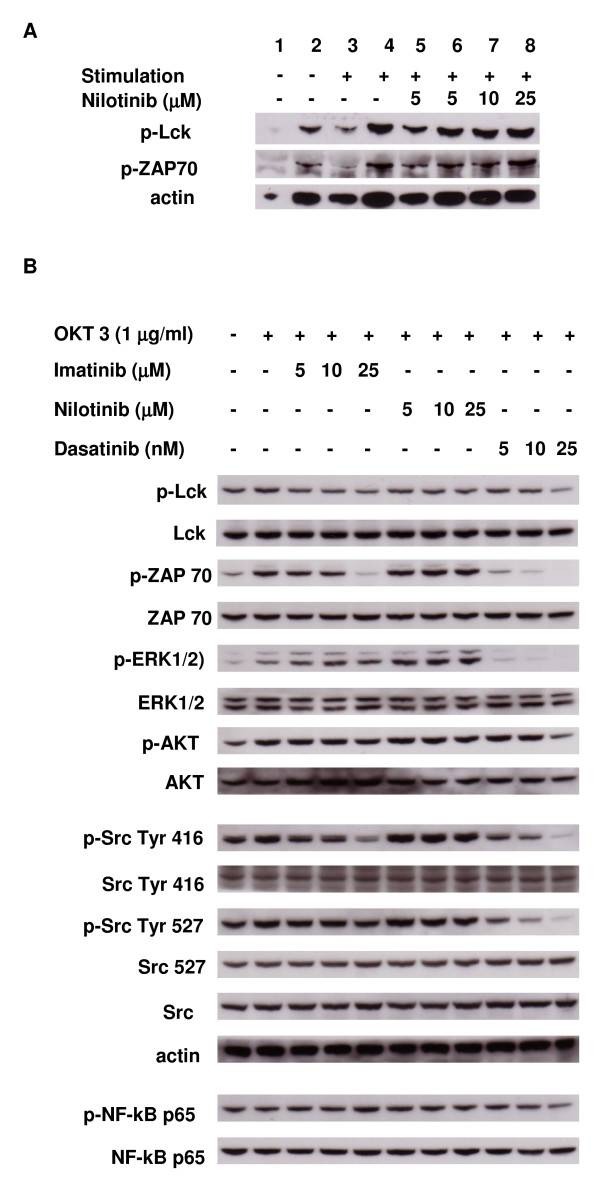
**Nilotinib does not inhibit the signaling events on TCR in CD4^+^CD25^+ ^T cells and CD4^+^CD25^- ^T cells**. **Panel A**: Purified CD4^+^CD25^+ ^T cells and CD4^+^CD25^- ^T cells were incubated with nilotinib for 1 hour. Then cells were not stimulated or stimulated with anti-CD3/CD28 for 15 minutes. Whole cell lysates were analyzed by western blotting for the phosphorylation levels of Lck and Zap70. (1. non stimulated CD4^+^CD25^+ ^T cells; 2. non stimulated CD4^+^CD25^- ^T cells; 3. stimulated CD4^+^CD25^+ ^T cells; 4. stimulated CD4^+^CD25^- ^T cells; 5. stimulated CD4^+^CD25^+ ^T cells plus nilotinib; 6. stimulated CD4^+^CD25^- ^T cells plus nilotinib; 7. stimulated CD4^+^CD25^- ^T cells plus nilotinib; 8. stimulated CD4^+^CD25^- ^T cells plus nilotinib; the respective concentration of nilotinib is indicated in the figure.) **Panel B**: Comparison of imatinib, nilotinib and dasatinib on the effects of TCR, Src and NF-κB signaling molecules in the Jurkat cell line. Compared with imatinib and dasatinib, nilotinib did not show obvious inhibitory effects in Jurkat T cells, while dasatinib showed the highest inhibitory potency among the three drugs. Data are representative of three experiments with similar results.

CD4^+^CD25^+ ^T cells and CD4^+^CD25^- ^T cells were incubated with or without different concentrations of nilotinib for one hour. After incubation, cells were stimulated with anti-CD3 and anti-CD28 for 15 minutes. Nilotinib did not significantly decrease the levels of p-Lck and p-ZAP even at a high concentration of 25 μM as shown in Figure 6A.

Furthermore, we compared the effects of nilotinib, imatinib and dasatinib on TCR, Src and NF-κB dependent signal cascades in Jurkat T cells. Figure 6B clearly shows that exposure of cells to low nanomolar concentrations of dasatinib attenuated the phosphorylation levels of Lck, ZAP-70, ERK 1/2, AKT, Src Tyr416, Src Tyr527 and NF-κB P65 in a dose-dependent manner. Imatinib only showed significant effects at a dose of 25 μM which is 100-fold higher concentrations than dasatinib. In contrast, nilotinib showed no significant inhibition of TCR, Src and NF-κB signal events, even at a high concentration of 25 μM.

## Discussion

The novel, selective Abl inhibitor nilotinib was designed to interact with the ATP-binding site of BCR-ABL with a higher affinity than imatinib. Besides being significantly more potent when compared with imatinib, nilotinib also maintains activity against most of the BCR-ABL point mutants that confer to imatinib resistance. In phase I/II clinical trials administration of nilotinib resulted in cytogenetic and hematologic responses in imatinib-refractory CML patients [2].

Now nilotinib represents an additional therapeutic option for patients with progressive CML [26].

Naturally occurring Tregs represent between 5% and 10% of the CD4^+ ^T cell subset in the peripheral blood of healthy volunteers [27,28]. Studies of T-cell mediated immunoregulation provide crucial insights into the immune system's task of balancing immunologic self-tolerance, while preserving tumor and anti-microbial immunity. Tregs have emerged as key cellular components that mediate this process [9]. In the stem cell transplantation setting, Tregs have proved to be effective in suppressing lethal graft versus host disease (GVHD). Importantly, this suppression does not abrogate the beneficial graft versus tumor (GVT) effect in most murine models. Moreover, in preliminary studies, donor Tregs promote engraftment and enhance immune reconstitution [9]. Recently, patients with CML are treated with nilotinib when the therapy with imatinib failed or caused serious side effects [2]. The same applies to the situation of CML patients after allogeneic stem cell transplantation. Moreover, patients with a history of nilotinib administration before transplantation are likely to be treated again by the drug in the case of a relapse of the disease after allogeneic stem cell transplantation. Therefore, the effect of nilotinib on Treg function needs to be monitored [9,29].

In an effort to investigate the potential role as nilotinib as an immunomodulatory agent, we set our studies on three important parts of T cell responses: TCR signaling and expression of activation markers, cytokine production, and proliferation [13]. In our study, we observed that therapeutic doses of nilotinib did not hamper the proliferation and function, of either CD4^+^CD25^+ ^T cells or CD4^+^CD25^- ^T cells. Nilotinib only showed significant inhibitory effect on CD4^+^CD25^+ ^T cells or CD4^+^CD25^- ^T cells at a concentration higher than 10 μM. However, Chen et al showed that nilotinib inhibits phytohemagglutinin (PHA)-induced proliferation of CD8^+ ^T cells *in vitro *at therapeutically relevant concentrations (0.5-4 μM) [16]. Similar results were also shown by Blake et al. [30]. We think the difference between us might be the reason we use anti-CD3, anti-CD28 and IL-2 to stimulate CD4^+^CD25^+ ^T cells and CD4^+^CD25^- ^T cells which is stronger than PHA that could partly abrogate the inhibitory effect of nilotinib on cells. However, the correlates between nilotinib and T cells *in vivo *are still unknown and it is still not clear which assays are more appropriate in gauging the suppressive effect of nilotinib. Furthermore, our results present that nilotinib did not affect the suppressive capacity of Tregs at therapeutically relevant concentrations, only at a concentration of 5 μM. Tregs and nilotinib act in synergy to reduce CD4^+^CD25^- ^T cells proliferation when co-cultured at the same time. We propose that the reason is that the direct inhibitory effect of nilotinib on CD4^+^CD25^- ^T cells might be stronger than the indirectly inhibitory effect on the proliferation of CD4^+^CD25^- ^T cells by Tregs.

The recent identification of the FoxP3, as a more specific marker of Tregs better defines the regulatory subset of CD4^+ ^T cells from activated effector T cells [9]. Based on consistent findings in mice and humans, FoxP3 is considered the "master regulator" of Tregs [9]. GITR is highly and constitutively expressed on the surface of mouse and human Tregs. Stimulation of GITR *in vitro *or *in vivo *or the removal of T cells expressing high levels of GITR leads to autoimmunity in normal mice [31,32]. In our study, we observed that high doses of nilotinib down-regulated the expression of FoxP3 and GITR in Tregs in a dose-dependent manner, which was accordance to the function of Tregs.

In the next step, we investigated the signaling events in CD4+CD25+ T cells and CD4+CD25- T cells after treatment with nilotinib. Consistent with previous data, nilotinib did not impair the phosphorylation levels of Lck and ZAP70 in cells even at a high concentration of 25 μM. Furthermore, we compared the inhibitory effects of imatinib, nilotinib and dasatinib on signal events against Jurkat T cells. An interesting phenomenon is that imatinib-resistant CML patients who develop resistance against nilotinib may still show a response to dasatinib, and patients with resistance against dasatinib may still respond to nilotinib [33]. Another remarkable aspect is that, in contrast to nilotinib, dasatinib exhibits a number of clinically relevant side effects including cytopenia and pleural effusions when applied at approved doses [34]. All these observations point to major differences of the three tyrosine kinase inhibitors regarding their mechanism of action and target profiles in pathological as well as normal cells [33]. We demonstrated that among the three drugs, dasatinib showed the highest potency on TCR, Src and NF-κB signaling events, while nilotinib did not show inhibitory effects on this signaling transduction cascade, which is in accordance with the molecular mechanisms of the three drugs. Previous biochemical studies have already revealed pronounced differences between the three tyrosine kinase inhibitors with regard to their selectivity. Nilotinib, like imatinib, inhibits BCR-ABL, c-ABL, c-KIT, and PDGFR, although with greater potency and selectivity for BCR-ABL [5]. The selectivity of nilotinib against BCR-ABL (relative to other targets, such as Src-family or c-Kit kinases) may account for the high level of efficacy unaccompanied by higher rates of severe myelosuppression [6]. Dasatinib, on the other hand, has been developed as a dual-specificity Abl- and Src-family kinase inhibitor [35]. Moreover, several pathways of the immune system could be severely affected by continuous high doses of dasatinib, harboring significant risks for immunosuppression of patients treated over a long period of time [33]. Recently, our group shows that imatinib inhibits the proliferation and function of Tregs and CD8^+ ^T cells as a concentration range of 1-5 μM, while the range for dasatinib is 5-10 nM [18,36,37]. Larmonier et al reported that imatinib inhibits the suppressive function and FoxP3 expression on Tregs as low as 1 μM. *In vivo *study indicated that imaitnib decreases Treg frequency and impairs their immunosuppressive function for mice treated with imatinib but imatinib does not impair the production of IL-10 and TGF-β *in vitro *[38]. Dasatinib proves to be much more potent than imatinib and nilotinib on Tregs and CD8^+ ^T cells. Chow et al. reported that although nilotinib exhibits only a minor apoptosis-inducing effect in the T-cell lines, it exerts a considerable, dose-dependent cytotoxicity in the B-cell lines. The activity of nilotinib is not restricted to Bcr-Abl, c-kit, or PDGFR-positive cells, but also extends to lymphatic cell lines of B-cell origin at a concentration of 5 μM [39]. Furthermore, Hipp et al. indicated that the multitargeted tyrosine serine/threonine kinase inhibitor sunitinib could significantly decrease the number of Tregs in the peripheral blood of mice treated with subtoxic doses of the drug, but does not show an impaired CD8^+ ^T cell response [40]. As all these compounds have already entered clinical practice, it would be useful to further define the appropriate clinical angle, because it would allow a rational approach to balance effector T cells and Tregs especially for patients after allogeneic stem cell transplantation.

In conclusion, our results show that the tyrosine kinase inhibitor nilotinib can inhibit both proliferation and function of Tregs and CD4^+^CD25^- ^T cells only at high concentrations, which exceeds therapeutically relevant concentrations of the drug. Since nilotinib has less inhibitory effects on Tregs than imatinib and dasatinib, nilotinib might constitute a good choice for patients in transplantation settings.

## Competing interests

The authors declare that they have no competing interests.

## Authors' contributions

FF carried out the molecular biology studies, participated in the sequence alignment and drafted the manuscript and carried out the immunoassays, YY carried out analysis the immunoassays and drafted the manuscript, AS participated in the design of the study, MTR carried out FACS, BC participated in the design of the study, JG participated in the design of the study, MG participated in the sequence alignment and helped to draft the manuscript, DB participated in its design and coordination and helped to draft the manuscript and MS conceived of the study, and participated in its design and coordination and finalized the manuscript. All authors read and approved the final manuscript.
